# Ethanol actions on the ventral tegmental area: novel potential targets on reward pathway neurons

**DOI:** 10.1007/s00213-018-4875-y

**Published:** 2018-03-16

**Authors:** Chang You, Bertha Vandegrift, Mark S. Brodie

**Affiliations:** 1 Department of Physiology and Biophysics, University of Illinois at Chicago, 835 S. Wolcott Ave, Room E-202, M/C 901, Chicago, IL 60612 USA; 2 Center for Alcohol Research in Epigenetics, Department of Psychiatry, University of Illinois at Chicago, Chicago, IL 60612 USA

**Keywords:** VTA, Epigenetic, Dopamine, Reward/aversion, Sex differences, Alcoholism

## Abstract

The ventral tegmental area (VTA) evaluates salience of environmental stimuli and provides dopaminergic innervation to many brain areas affected by acute and chronic ethanol exposure. While primarily associated with rewarding and reinforcing stimuli, recent evidence indicates a role for the VTA in aversion as well. Ethanol actions in the VTA may trigger neuroadaptation resulting in reduction of the aversive responses to alcohol and a relative increase in the rewarding responses. In searching for effective pharmacotherapies for the treatment of alcohol abuse and alcoholism, recognition of this imbalance may reveal novel strategies. In addition to conventional receptor/ion channel pharmacotherapies, epigenetic factors that control neuroadaptation to chronic ethanol treatment can be targeted as an avenue for development of therapeutic approaches to restore the balance. Furthermore, when exploring therapies to address reward/aversion imbalance in the action of alcohol in the VTA, sex differences have to be taken into account to ensure effective treatment for both men and women. These principles apply to a VTA-centric approach to therapies, but should hold true when thinking about the overall approach in the development of neuroactive drugs to treat alcohol use disorders. Although the functions of the VTA itself are complex, it is a useful model system to evaluate the reward/aversion imbalance that occurs with ethanol exposure and could be used to provide new leads in the efforts to develop novel drugs to treat alcoholism.

## Ethanol actions on the ventral tegmental area

### Why focus on the ventral tegmental area for therapeutics?

Focusing on any one brain area with a goal of development of therapeutic agents is fraught with complications, as different brain areas are likely to react differently to any single drug. However, work over the past 30 years has clearly delineated circuitry involved in different aspects of addiction (Koob and Volkow 2016). Like an electrical circuit, interruption of a key point of the brain pathways involved in the expression of symptoms of alcohol addiction may be sufficient to disrupt the aspects of addiction (craving, for example) that prevent the maintenance of sobriety. Because of its importance as the “headwaters” of dopamine in reward circuitry and because of the importance of dopamine in reward, reinforcement, memory, and cognition, the ventral tegmental area (VTA) is a rational point to examine changes in brain physiology induced by alcohol exposure and, in addition, serves as a useful model system for investigations into neuroadaptation to alcohol and possible ways to ameliorate these changes. When dealing with circuits like those involved in alcohol and drug addiction (Koob and Volkow 2010), the therapeutic goal is to intervene at a point that relieves the greatest number of relevant symptoms while producing the fewest deleterious side effects.

The ventral tegmental area is known to be important in the mediation of reward and reinforcement of drugs of abuse (Lammel et al. 2014; Ranaldi 2014) and natural reinforcers like food (Meye and Adan 2014). While changes in the VTA induced by alcohol exposure may precede alcohol-induced alteration in other brain areas (Koob and Volkow 2010), the role of dopamine may continue to be significant throughout the course of alcohol use disorders. In addition to their importance in the nucleus accumbens (NAc) and prefrontal cortex (PFC) for reward and evaluation of salience, dopaminergic (DA) projections from the VTA are crucial in other brain areas such as amygdala (Lee et al. 2016), prefrontal cortex (Kroener et al. 2009), and hippocampus (Navakkode et al. 2012), to facilitate physiological functions related to emotional memory. Disruption of dopamine signaling may be an early event that leads to, or exacerbates, pathology in other brain regions (Koob and Volkow 2010). Furthermore, restoration or amelioration of dopaminergic neurotransmission may help disrupt pathological adaptations to chronic alcohol exposure and withdrawal, as is beginning to be examined in other addictive disorders (O’Neill et al. 2014; Perez et al. 2015; Wu et al. 2014). Most of the studies cited below represent preclinical studies. Preclinical studies in animal models can yield important information, but human disease is more complex than any single model. Disruption of circuits in animals may produce a decrease in drinking behavior, for example, but the complexity of human behavior may circumvent one particular circuit in favor for a different one. Examination of a region like the VTA, with its early involvement in processes leading to addiction (Koob and Volkow 2010) and extensive connections to numerous brain areas, may be more productive than looking at brain regions primarily involved in emotion or memory. While the VTA may not be the sole brain area subject to pathological adaptation due to alcohol abuse (it is clear that many brain areas are affected), focusing on the VTA for therapy for alcoholism may be prudent due to its importance in reward/reinforcement circuitry and as a model system in which the actions of a variety of agents can be tested.

### Balance between reward and aversion

In addition to the well-established mediation of reward and reinforcement, recent studies suggest another role for the VTA in communicating the aversive nature of stimuli (Juarez and Han 2016). The VTA receives GABAergic inhibitory input not only from NAc but also from the rostromedial tegmentum (RMTG), which in turn receives input from the lateral habenula (LH); there is also a direct connection between the LH and the dopamine neurons of the VTA (Lammel et al. 2012). While the connection from lateral VTA neurons to the NAc is important for reward, there is also a projection from the more medial VTA to the prefrontal cortex that is involved in signaling related to aversion (Lammel et al. 2014). The complexity of different sub-regions of the VTA has added some confusion to the literature, but simply put, the reward neurons of the VTA (mainly the lateral dopaminergic neurons that project to the NAc) are activated by rewarding stimuli and inhibited by aversive stimuli (Juarez and Han 2016). The arguments in this review are based on this simplification, but, in fact, there is evidence that a small subpopulation of VTA neurons are activated by aversive stimuli (Lammel et al. 2014; Schultz 2010). Examination of neuroadaptation in the VTA related to ethanol exposure should take into account these two functions of the VTA and accept the premise that the balance between aversion and reward signaling of the VTA may play a role in driving alcohol seeking.

### Overview of the VTA

The complexity of the functions of the VTA is reflected in the diversity of cellular composition and projection targets. The VTA is composed of a heterogeneous collection of cells. In addition to being rich in tyrosine hydroxylase (TH)-positive dopaminergic neurons, the VTA is also composed of GABA and glutamatergic neurons (Lammel et al. 2014). GABAergic neurons of the VTA, which can be identified by the expression of glutamic acid decarboxylase (GAD) (Margolis et al. 2012), can be further classified as interneurons or neurons that project to the prefrontal cortex, ventral palladium, lateral hypothalamus, or lateral habenula (Carr and Sesack 2000; Taylor et al. 2014). Phenotypic diversity of VTA neurons may be compartmentalized into anatomical regions. The five regions of the VTA (rostral linear nucleus of the raphe, interfascicular nucleus, caudal linear nucleus, paranigral nucleus, and parabrachial pigmented nucleus (Oades and Halliday 1987)) may be simplified into “medial” and “lateral” VTA when considering phenotypic organization such as responsivity to ethanol (Morales and Margolis 2017). Populations of DA neurons in the medial VTA are more sensitive to ethanol than those in the lateral VTA (Mrejeru et al. 2015).

GABAergic and glutamatergic afferents to the VTA appear to play important roles in coordinating the effects of drugs of abuse. GABAergic innervation to the VTA originates from many brain regions including the rostromedial tegmental nucleus (RMTg), lateral habenula, ventral palladium, nucleus accumbens, and amygdala (Janak and Tye 2015; Jhou et al. 2009; Rahman and McBride 2000). The inhibitory RMTg GABA input to VTA DA neurons plays a role in regulating aversion (Jhou et al. 2009). The ventral pallidum regulates motivation by providing inhibitory control to DA and non-DA VTA neurons (Hjelmstad et al. 2013). The lateral hypothalamus and bed nucleus of stria terminalis (BNST) project both GABAergic and glutamatergic input onto the VTA (Geisler et al. 2007; Jalabert et al. 2009; Kallo et al. 2015). The BNST, which plays a role in responding to fear and stress, directly regulates the GABA and DA neurons of the VTA and indirectly inhibits DA VTA neurons by disinhibiting local GABA neurons in the VTA (Kudo et al. 2012). Glutamatergic neurons from the BNST innervate GABA neurons of the VTA to regulate behaviors related to aversion (Jennings et al. 2013). The laterodorsal tegmental nucleus (LDT) is a source of GABAergic, glutamatergic, and cholinergic input to the VTA (Wang and Morales 2009). The LDT generally exerts an excitatory control over VTA DA neurons (Lammel et al. 2012; Lodge and Grace 2006). During different stages of alcohol dependence, neuroplasticity in these brain areas will influence their regulation of the VTA (Koob and Volkow 2010) and could modulate VTA activity during alcohol use and withdrawal.

### Disruption of dopamine transmission and drinking behavior.

The importance of the ventral tegmental area and dopaminergic neurotransmission in the establishment and maintenance of alcohol intake in animals has been a topic of study for at least 30 years. The seminal studies of Pfeffer and Samson (1988; Samson et al. 1988a, b) showed that drugs acting on dopaminergic systems disrupted alcohol drinking. Interestingly, both dopamine agonists and antagonists reduce ethanol intake (Pfeffer and Samson 1988). The pattern of intake differs between rats treated with a dopamine agonist compared to those treated with a dopamine antagonist; the agonist induces a pattern similar to satiety, and the antagonist induces a pattern more similar to extinction (Pfeffer and Samson 1988). More evidence supporting the role of dopamine in ethanol reward shows that acute ethanol increases the release of dopamine in the nucleus accumbens (Imperato and Di Chiara 1986; Vena and Gonzales 2015) and prefrontal cortex (Schier et al. 2013). Furthermore, delivery of ethanol directly into the VTA supports self-administration (Rodd et al. 2004a; b). From a therapeutic perspective, the acute actions of ethanol on the dopaminergic system, mechanisms of ethanol action, and regulation of those mechanisms by other processes, are important to better understand mechanisms of adaptation to ethanol after chronic alcohol use, and to understand the sequence of molecular events leading from reward-seeking to compulsive use.

### Effects of ethanol on receptors and ion channels in the VTA and relevance for therapeutics:

Ethanol activates neurons of the VTA in vivo (Diana et al. 1992a; Gessa et al. 1985) and in vitro (Brodie et al. 1990, 1999). A superb review of alcohol effects in the VTA (Morikawa and Morrisett 2010) carefully examined the actions of ethanol on neurons of the VTA and provided a comprehensive assessment of the literature regarding the effects of a variety of agents on ethanol activation of VTA neurons. There is no need to recapitulate their analysis here.

**Fig. 1:**
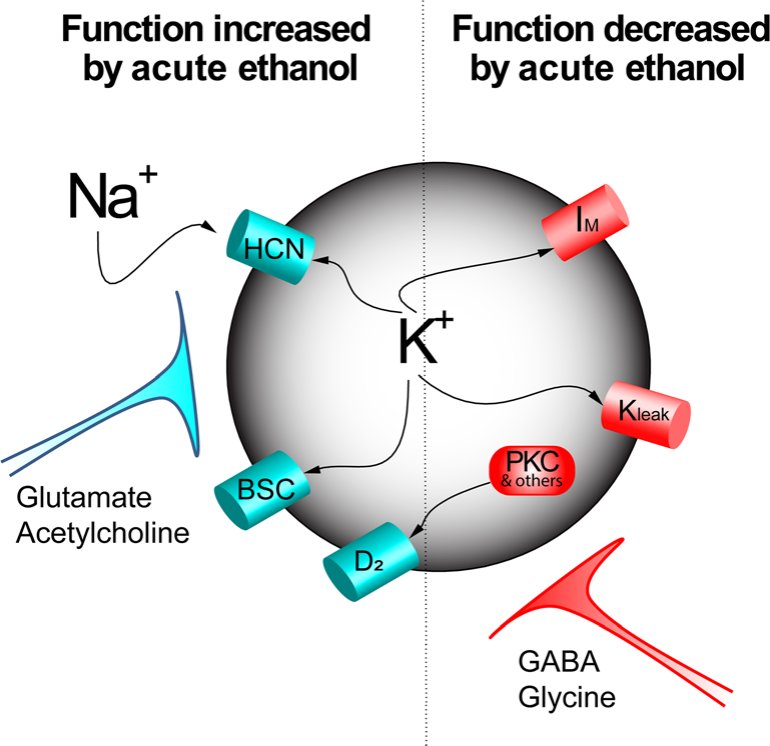
Numerous effects of ethanol have been demonstrated on VTA neurons. Acute ethanol increases the mixed Na^+^/K^+^ h-current (HCN) and also increases a barium-sensitive current (BSC). Acute ethanol also decreases m-current (*I*_*M*_) and a leak potassium channel (*K*_leak_) that contributes to increased excitability. Acute ethanol also decreases D_2_ dopamine receptor desensitization (which would have the effect of increasing dopamine inhibition), possibly by reducing the activity of protein kinase C or other elements of the desensitization pathways (PKC and others). In addition to the direct actions of ethanol on the membrane of VTA neurons, there can also be significant influences of afferents to the VTA, some of which are increased (glutamate and acetylcholine) and some of which are reduced (GABA, glycine). The sum of these effects results in the ethanol-induced excitation and increased release of dopamine to target areas; chronic alcohol exposure can result in differential tolerance to one or more of these effects, altering the tonic activity of VTA neurons (Diana et al. 1992b; Shen and Chiodo 1993) and increasing the rewarding value of ethanol (Brodie 2002; Hopf et al. 2007; Rodd et al. 2005)

A number of non-synaptic targets of ethanol have been identified, indicating that alcohol can act directly on VTA neurons. Among these are effects which appear to be both excitatory and inhibitory (Fig. 1). Ethanol increases hyperpolarization-activated current (h-current) (Brodie and Appel 1998; Okamoto et al. 2006) and reduces m-current (Koyama et al. 2007); both effects may excite VTA neurons. In contrast, ethanol increases barium-sensitive potassium currents in the VTA (McDaid et al. 2008), as has been shown in cerebellum (Lewohl et al. 1999), an effect that should inhibit VTA neurons. Chronic ethanol increases the excitatory response of VTA neurons to acute ethanol administration (Brodie 2002), suggesting some specific adaptation that occurs with repeated ethanol exposure. This adaptation could be an increase in the excitatory effects of ethanol or a decrease in the inhibitory effects. A shift in the balance of ethanol actions from aversion and toward reward may underlie the specific neuroplasticity within the VTA that increases drug-seeking behavior (Pignatelli and Bonci 2015), including alcohol seeking. For this reason, pharmacotherapy targeting the rewarding actions of ethanol may be less effective unless the aversive effects can be restored as well.

With respect to the specific targets of ethanol responsible for the activation of VTA neurons, some general comments should be made regarding the variety of neurotransmitters and ion channels that have been suggested to play an important role. While acute action of ethanol on neurotransmitter action or ion channel function is vital to our understanding of ethanol action on dopaminergic neurotransmission, these actions become more relevant in examination of changes induced by chronic alcohol. Numerous neurotransmitters have been suggested to play a direct or indirect role in ethanol-induced activation, including GABA (Gallegos et al. 1999; Stobbs et al. 2004; Xiao and Ye 2008), glutamate (Deng et al. 2009; Xiao et al. 2009), glycine (Ye et al. 2001), and acetylcholine (Clark and Little 2004; Ericson et al. 2008). For example, several labs have focused on GABAergic systems, and disinhibition of dopaminergic (DA) VTA neurons by intrinsic GABA neurons or by GABAergic inputs from the RMTG (Fu et al. 2016; Melis et al. 2014) or NAc (Juarez and Han 2016). Alcohol effects on a variety of other ion channels and neurotransmitter receptors in the VTA or the action of antagonists of those receptors or ion channels have been reported and are well described in the review article cited above (Morikawa and Morrisett 2010). While these studies illuminate the variety of systems affected by ethanol, they do not address the primary response to ethanol, i.e., increased VTA neuron firing rate and increased dopamine release. However, ethanol excitation of DA VTA neurons is not diminished by dissociating the neurons from synaptic and glial connections (Brodie et al. 1999), nor by the presence of antagonists of GABA_A_, GABA_B_, NMDA, AMPA, metabotropic glutamate, muscarinic or nicotinic cholinergic receptors (Nimitvilai et al. 2016), indicating that these receptors are not necessary for the excitatory action of ethanol on DA VTA neurons in vitro. Although the specific target of ethanol action responsible for excitation of DA VTA neurons has not been identified, from a therapeutic standpoint, it is not necessary that a receptor or ion channel be the primary target of ethanol to have an important influence on the rewarding action of alcohol. Neurotransmitters impinging on VTA neurons may alter membrane electrical properties and interfere with transmission of an ethanol-specific signal or all signals. Once the primary specific target(s) of ethanol action in the VTA are identified, the effects of other agents on the modulation of ethanol-sensitive signal by ion channels and neurotransmitter receptors can be placed in the appropriate context. For example, G protein-coupled potassium channel 3 (GIRK3) has been proposed as an important regulator of ethanol sensitivity in the VTA (Herman et al. 2015) but earlier evidence that antagonists of GIRKs fail to affect ethanol excitation of VTA neurons (Appel et al. 2003; McDaid et al. 2008) indicates that GIRK3 is not the primary target of alcohol action in the VTA. Despite this fact, specific regulators of GIRK3 activity may become useful modulators of ethanol action on the reward pathways. Likewise, selective blockade of h-channels in the VTA could reduce ethanol activation of the VTA (Okamoto et al. 2006) by increasing the efficacy of an inhibitory effect of ethanol (activation of barium-sensitive currents) without altering the excitatory effect (McDaid et al. 2008), providing another avenue for regulating ethanol action. This type of novel strategy could be an effective approach in restoring the reward/aversion balance of ethanol action in the VTA.

### Focus on adaptive mechanisms

In addition to these relatively “static” effects on VTA neurons (mechanism, or modulation, of ethanol-induced excitation), more dynamic processes also may be affected by chronic or acute ethanol. These processes include synaptic plasticity and receptor trafficking. Long-term potentiation (LTP) and long-term depression (LTD) of dopamine (Hausknecht et al. 2017; Wanat et al. 2008) and GABA neurons (Guan and Ye 2010) in the VTA is reduced by prior in vivo ethanol exposure. As a model of rapid synaptic remodeling in response to a brief event, ethanol-induced changes in LTP may be useful as a model for examination of ethanol actions on memory processes related to stimulus salience (Schultz 2010). Furthermore, LTD and LTP in the reward pathways may be mechanisms during the addiction process by which the signaling of aversive properties of stimuli are reduced and the rewarding properties are enhanced (Pignatelli and Bonci 2015).

Likewise, a fundamental physiological process like desensitization of G protein-coupled receptors (GPCRs) may be very relevant to alteration of VTA neuronal physiology by alcohol use. Desensitization of the D_2_ dopamine receptor on VTA neurons is impaired in the presence of low concentrations (10–25 mM) of ethanol (Nimitvilai et al. 2012a), even though the initial response to dopamine is unchanged in the presence of ethanol. Complex regulation of the desensitization process may include more than one ethanol-sensitive component, including protein kinase C (Fig. 1), G protein-coupled receptor-associated sorting protein (GASP) (Bartlett et al. 2005), G protein-coupled receptor kinase 2 (GRK2) (Nimitvilai et al. 2013), and anaplastic lymphoma kinase (ALK) (Dutton et al. 2016). Other neurotransmitter systems participate in the mechanism of D_2_ dopamine receptor desensitization, including glutamate and CRF receptors (Nimitvilai et al. 2014), and there is cross-desensitization between D_2_ receptors and other GPCRs (Nimitvilai et al. 2012b, 2013). While dynamic cellular processes like desensitization are generally more difficult to study, they might be more relevant in the context of behaviors related to alcohol reward and alcohol seeking. Amelioration of the effects of ethanol on one or more of these processes may restore the aversion/reward balance to the actions of alcohol.

The mechanisms of both LTP and D_2_ receptor desensitization are complex, but the pathways for these processes are under extensive study, and as they are elucidated, additional components that regulate and modulate these processes become more apparent and these regulatory mechanisms may be more amenable to therapeutic intervention. As mentioned above, one example is the molecule ALK, a receptor tyrosine kinase that is associated with alcohol dependence in humans. In a mouse model, ALK antagonists TAE684 and alectinib not only reduced binge alcohol drinking but they also impair D_2_ receptor desensitization (Dutton et al. 2016). The interaction between the D_2_ desensitization pathway and ALK action is unknown, but may offer a novel approach to therapy.

Other signaling pathways that may provide fertile avenues for development of alcohol treatment therapies are the proto-oncogene tyrosine-protein kinase FYN and HRAS pathways that have been very well investigated by the Ron laboratory (Ron and Barak 2016). Exposure to alcohol and repeated withdrawal leads to upregulation of HRAS and FYN activity. Both of these pathways play a role in processes associated with learning and memory and are also linked to synaptic plasticity (Kaneko et al. 2010; Ohnishi et al. 2011). In brain areas that are targeted by the VTA such as the NAc and prefrontal cortex, HRAS may affect desensitization (via G protein-coupled receptor activation) and FYN is associated with enhancement of LTP/LTD (via alteration of glutamatergic receptor function). Action on regulatory agents such as ALK and FYN may be a more subtle, and potentially more successful, way to approach alcohol influences on dynamic processes, and ultimately may be better therapeutic targets than dopamine or glutamate receptors per se.

Although there is now an extensive catalog of agents that alter ethanol responses of VTA neurons, single-receptor or single-ion channel pharmacology may have significant limits in terms of the development of therapeutic agents. Altering the efficacy of GABAergic transmission, or alteration of GIRK3 function, in the VTA may alter alcohol responses, but systemically administered agents that affect these targets seem likely to produce a host of undesirable side effects, as both GABA receptors (Billinton et al. 2000; Waldvogel and Faull 2015) and GIRK3 channels (Saenz del Burgo et al. 2008) are prevalent throughout the nervous system.

All of these cellular phenomena are focused on the acute actions of ethanol, or adaptation to chronic ethanol exposure, in the VTA. Ultimately, the goal would be to address the underlying mechanisms induced by chronic ethanol treatment to normalize brain physiology and restore the reward/aversion balance in the VTA. With the idea that the changes in the brain induced by chronic ethanol intake are long-lasting and sustained even after long periods of abstinence (Seo and Sinha 2014), an approach at the genomic level may be a more effective strategy for development of therapeutically useful agents.

## Epigenetic actions of ethanol: a novel avenue for treatment

In general, adaptive changes in the brain produced by alcohol are long lasting; those brain changes that promote alcohol seeking outlast acute withdrawal and can undermine sobriety years after the individual has stopped drinking. Recent developments in the study of interactions between the environment and the genome point to epigenetics as underlying the nature of those alcohol-induced changes. Approaching therapy for alcoholism from the standpoint of the epigenome may be an effective way to reverse alcohol-induced brain changes as an adjunct to other treatments for alcoholism. While the application of epigenetics methodology to alcohol and substance use disorders is relatively recent, the hope is that overcoming alcohol-induced changes in brain physiology at the level of the epigenome will restore the reward/aversion balance in the VTA, and in other brain areas.

Epigenetics can be broadly defined as alterations of gene information, without affecting the DNA sequence (Holliday 2006), and is an emerging area in the fields of alcohol and substance abuse (Nestler 2013; Pandey et al. 2008). Although genetic factors can be reliable predictors of risk for some diseases, for psychiatric disorders like alcoholism it is commonly accepted that genes themselves account for only about 40–60% of the variance involved in alcoholism (Prescott and Kendler 1999). Some “inheritable” factors of alcoholism are now suggested to be epigenetic-based. For example, parental exposure to ethanol prior to conception, via epigenetic mechanisms, can affect ethanol preference and sensitivity, stress phenotype in offspring and gene expression in VTA (Finegersh and Homanics 2014; Rompala et al. 2017). Social environmental elements also affect the vulnerability to addiction to drugs such as alcohol (Ajonijebu et al. 2017). Growing evidence suggests that epigenetic modifications play critical roles in brain changes induced by drugs of abuse (Nestler 2013), including alcohol (Farris et al. 2015b; Krishnan et al. 2014; Kyzar and Pandey 2015; Moonat and Pandey 2012; Ponomarev et al. 2012; Starkman et al. 2012). From the perspective of the VTA, long-term alteration in function following ethanol exposure, such as changes in GABA sensitivity, may be determined by epigenetic modifications (Arora et al. 2013). Reversing those modifications may restore pre-exposure function and may support abstinence over relapse.

**Fig. 2:**
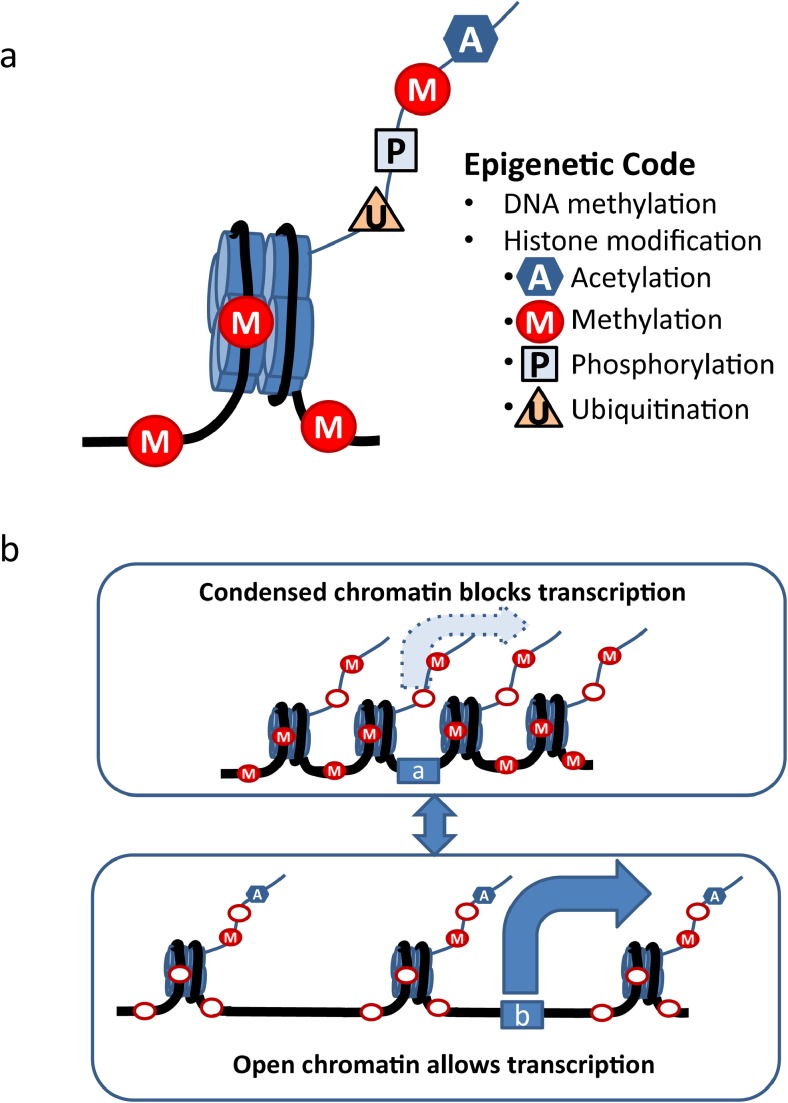
**a** Modifications that compose the “epigenetic code.” DNA methylation adds methyl groups onto the cytosine residues on the CpG islands of the DNA strand. Another group of modifications are histone modifications, which make up the tails of histone molecules around which the DNA strand wrap. There are four well-established types of histone modification, which are acetylation, methylation, phosphorylation, and ubiquitination, that can increase or decrease transcription, depending on the site, the number, and the type of modification. The sum of these modifications determines the net effect on gene transcription. **b** DNA methylation is believed to silence or repress the transcription of DNA information. On the other hand, histone acetylation generally promotes gene transcription. Top diagram: Methylation (M) of the DNA and a lack of acetylation (A) on histone tails usually causes condensation of chromatin, blocking gene transcription (a). Bottom diagram: DNA demethylation (open red ovals) and histone acetylation (A) can lead to opening of the chromatin and gene transcription (b). Histone methylation can have a more complex effect on transcription. The number and location of sites on the histone that are methylated (note different sites methylated and demethylated on the histone tail in the top and bottom diagrams) can exert different influences on the chromatin structure. Histone phosphorylation and ubiquitination are also complex mechanisms, and the effects of alcohol on these processes are not well understood

The most commonly accepted epigenetic mechanisms can be categorized into DNA methylation, histone modification, noncoding RNA (ncRNA)-mediated and prion protein-mediated regulation (Bonasio et al. 2010; Choudhuri 2011; Houri-Zeevi and Rechavi 2017). Research into these last two types of epigenetic regulation are emerging but less is currently known with respect to alcohol and alcoholism and will not be examined in depth in this review. Significant evidence has accumulated thus far that DNA methylation, histone acetylation and histone methylation are associated with alcoholism. Briefly, chromatin can be divided into units called nucleosomes that are composed of histone octamers (two copies each of H2A, H2B, H3, and H4) and the DNA that wraps around them. DNA can undergo direct methylation, while histone modifications (acetylation, methylation, phosphorylation, and ubiquitinization) occur on histone “tails.” Combinations of different modifications of DNA and the histone tails determine the state of the chromatin to be either “open” (favoring gene transcription) or “condensed” (making gene transcription less likely) (Fig. 2). DNA methylation is believed to silence or repress the transcription of DNA information. On the other hand, histone acetylation generally promotes gene transcription. Methylation of DNA and lack of acetylation on histone tails usually results in condensation of chromatin, blocking gene transcription. DNA demethylation and histone acetylation can lead to the opposite effect, opening the chromatin for gene transcription. Histone methylation is a more complex epigenetic modification: the number (mono-, di-, or trimethylation) and location of histone methylation can exert different influences on the chromatin structure. Histone phosphorylation and ubiquitination are also complex mechanisms and their roles in the epigenetics of alcohol are less understood.

### Histone modifications in alcoholism

One major category of epigenetic regulation of gene expression is the histone modification. Chromatin is composed of DNA wrapped around histone octamers (Jenuwein and Allis 2001). Lysine residues on histone tails are the common location of histone modifications. The configuration of chromatin alterations affects the accessibility of transcription factors and other proteins to the DNA, therefore regulating gene transcription (Guertin and Lis 2010). In contrast to histone modification, the main epigenetic modification made directly to the DNA is methylation. However, it is important to acknowledge that DNA methylation and histone modifications are not solo players but rather interact and influence each other in the epigenetic regulation process (Rose and Klose 2014).

A global gene expression study on alcohol-preferring mice in a binge-drinking paradigm revealed that among genes in DA neurons that were differentially expressed between alcohol and control groups, the majority of the innately overexpressed genes in VTA DA neurons of alcohol-preferring mice were downregulated after binge drinking, and furthermore, expression of these genes were negatively correlated with ethanol consumption. Among the overrepresented functional groups coming out of this analysis were a cluster of genes involved in chromatin reorganization and modification, including histone acetyltransferases (HATs) and histone deacetylases (HDACs) (Marballi et al. 2016). Currently, most of the evidence in the VTA points to the importance of histone acetylation; other forms of epigenetic changes are less well studied in the VTA, but may have critical roles to play as well.

### Histone acetylation

The level of acetylation on histone tails of the chromatin is positively correlated with gene expression (Smith 1991). Histone acetyltransferases and deacetylases play critical roles in maintaining gene expression homeostasis in the brain. Restoring gene expression to non-pathological balance by targeting histone acetylation is a possible avenue for treatment of many psychiatric diseases, including drug addiction (Kazantsev and Thompson 2008).

Pharmacologically blocking innately high HDAC activities in alcohol-preferring (P) rats reduces anxiety-like and alcohol drinking behaviors (Moonat et al. 2013; Sakharkar et al. 2014). Similarly, HDAC inhibition attenuates anxiety-like and drinking behaviors during withdrawal after chronic ethanol exposure (You et al. 2014). Pharmacological HDAC inhibition diminished binge-like drinking behavior in mice (Warnault et al. 2013). Operant alcohol drinking was reduced by HDAC inhibition (Jeanblanc et al. 2015; Legastelois et al. 2013; Simon-O’Brien et al. 2015). The behavioral changes in these studies were seen using systemic administration of HDAC inhibitors (either by intraperitoneal or intracerebroventricular injection), so numerous brain regions would be affected and might be responsible for the behavioral response to HDAC inhibitors. Focusing on the VTA, our lab has shown that withdrawal from chronic ethanol is associated with hyposensitivity of VTA dopaminergic neurons to GABA (Arora et al. 2013; Brodie 2002). A similar change has been observed in amygdala, NAc, and cortex (Kumar et al. 2003; Papadeas et al. 2001). GABA hyposensitivity was associated with increased HDAC2 and decreased acetylation on histone 3, lysine 9 (H3K9) in the VTA (Arora et al. 2013). Expression levels of HDAC2 and acetylated histone protein in the VTA were equivalent to alcohol-naive controls in mice treated chronically with ethanol when ethanol was administered 1 h before sacrifice; this reversal of changes in epigenetic factors in the presence of ethanol suggests that after chronic ethanol treatment, ethanol is needed for “normal” VTA responses. A decrease in the α1 subunit of the GABA receptor in VTA was observed during withdrawal (Arora et al. 2013), but not after chronic ethanol treatment with no withdrawal (Arora et al. 2013; Papadeas et al. 2001); similar epigenetic regulation of GABA subunits in cultured cortical neurons has recently been reported (Bohnsack et al. 2017). Decreased GABA responsiveness in the VTA during withdrawal would alter signal processing in the VTA and the subjective evaluation of stimuli salience; normal responses would only be seen in the presence of ethanol.

Histone acetylation is also associated with the effects of other drugs of abuse in the VTA. HDAC inhibition also reversed morphine-induced changes in DA VTA activity (Authement et al. 2016). The volatile organic solvent toluene is another addictive drug that shares physiological effects with other drugs of abuse, including alcohol (Nimitvilai et al. 2016). Repeated toluene exposure altered acetylation of H3 in NAc and H4 in the VTA (Sanchez-Serrano et al. 2011). In the VTA of morphine-conditioned rats, histone H3 acetylation in the promoter region of *Dlg4* is associated with increase in the phosphorylated form of cyclic AMP response element binding protein (pCREB) binding to the *Dlg4* promoter region. Inhibition of pCREB activity in the VTA of these morphine-conditioned rats reversed these changes and enhanced reward behavior (Wang et al. 2014). Different substance abuse disorders may share some common mechanisms that alter chromatin, and interventions focusing on histone acetylation might be effective means of reversing molecular deficits related to addiction.

Compared to histone acetylation, investigations into other epigenetic modifications in the VTA induced by alcohol have been more limited. Other mechanisms that are currently being studied in connection with alcohol-induced epigenetic changes are histone methylation and DNA methylation.

### Histone methylation

Histone methylation is another form of chromatin modification. Histone methyltransferases (HMTs) transfer methyl groups from S-adenosylmethionine (SAM), onto histone N-terminal tail lysine or arginine residues. Histone demethylases (HDMs), which remove the methyl groups, are the counterpart of HMTs. Histone tail residues can be mono-, di-, or trimethylated; depending on the numbers of methyl groups and the location of these methylations, the biological effect can be very different. For instance, the mono-/trimethylation of histone H3K4, as well as mono-methylation of histones H3K9 and H3K27 are associated with upregulation of gene expression; while di-/trimethylation of H3K9 and H3K27 repress expression (Krishnan et al. 2014; Pattaroni and Jacob 2013; Strahl and Allis 2000) .

In human alcoholic brain, HMTs (MLL, MLL4, and SETD1A) that specifically trimethylate histone 3 lysine 4 (H3K4me3) were significantly upregulated (Ponomarev et al. 2012). Interestingly, global trimethylation and H3K4 trimethylation level was also upregulated in alcoholic human brains (Ponomarev et al. 2012). Cluster analysis from whole-genome sequencing of H3K4me3 in hippocampus from postmortem brain of alcohol-dependent individuals demonstrated that transcripts of genes in 83% of the modules were correlated with H3K4 trimethylation alteration (Farris et al. 2015a). Multiple polymorphisms in an HDM gene known as *Kdm4c* are associated with alcohol withdrawal symptoms (Wang et al. 2012). A ChIP sequencing study on alcoholic hippocampus indicated genome-wide changes in histone H3K4me3 (Zhou et al. 2011) and altered expression of histone deacetylases HDAC2 and HDAC4 (Zhou et al. 2011). Additional studies are needed to link histone methylation with the regulation of specific genes related to alcohol use disorders. Few studies have examined the involvement of histone methylation specifically in the VTA during alcoholism. However, it has been shown that histone methylation at *Bdnf* promoters II and III is reduced in the VTA during morphine abuse (Mashayekhi et al. 2012), suggesting that histone methylation is dynamically regulated in the VTA by drugs of abuse.

### DNA methylation

DNA methylation is catalyzed by DNA methyltransferases (DNMTs), a modification of DNA that involves adding a methyl group from SAM to the cytosine residues in the dinucleotide sequence CpG (Bestor 2000; Klose and Bird 2006). Transcription can be repressed by cytosine methylation of promoters, enhancers, and transcription start sites (Wolffe and Matzke 1999).

DNA methylation is involved in the mechanism of alcoholism as shown in both human and animal models (Tulisiak et al. 2017), but the studies to date suggest that both hypomethylation (Philibert et al. 2012) and hypermethylation (Manzardo et al. 2012) can be observed in postmortem alcoholic human brains. Whole-genome methylation profiling in the prefrontal cortex also found hypermethylated CpGs in male but not female alcoholic subjects (Wang et al. 2016), adding the complexity of sex differences to understanding the roles of DNA methylation in alcoholism.

In the VTA, changes in DNA methylation of specific genes is associated with reward-related associative memory (Day et al. 2013), which is essential for adaptation in alcohol addiction and substance use disorders. Studies have shown that the suppressed gene expression can be reversed by pharmacological approaches that can restore normal neuronal activity and drinking behaviors. Decitabine, an FDA-approved DNMT inhibitor, has been shown to accelerate desensitization to ethanol excitation of VTA dopaminergic neurons and to decrease ethanol intake (Ponomarev et al. 2017); these results are consistent with a reduction in ethanol salience produced by the DNMT inhibitor. Similar reductions of drinking were also observed using other DNMT inhibitors such as azacitidine (Warnault et al. 2013), and RG108 (Barbier et al. 2015). These observations suggest important roles of DNA methylation in reward circuitry in drug addiction and may suggest possible involvement in VTA-regulated reward and reinforcement.

### Epigenetic-based therapeutic agents and strategies

In light of the above observations, epigenetic mechanisms may serve as a focus of medication development for treatment of alcoholism. Examination of alcohol-induced epigenetic changes in the VTA provides useful information about neuroadaptation that occurs early in the time course of addiction and dependence.

The field of treatment for alcohol use disorders is in need of a novel pharmacotherapeutic approach. Currently, limited pharmacotherapy is available for the treatment of alcoholism as an adjunct to behavioral and psychological interventions. Medications that have been approved by the US Federal Drug Administration for the treatment of alcohol use disorders include naltrexone (opioid receptor antagonist), disulfiram (aldehyde dehydrogenase (ALDH) inhibitor), and acamprosate (NMDA glutamate receptor antagonist) (Ajonijebu et al. 2017). Disulfiram, in addition to ALDH inhibition, inhibits DNMT1 and reduces global 5-methylcytosine content (Lin et al. 2011). Naltrexone has not been shown to directly affect epigenetic regulation; however, its target opioid receptors are subject to epigenetic regulation. For example, reduction of μ-opioid receptor gene expression was associated with DNA hypermethylation in VTA, NAc, and PFC (Vucetic et al. 2011). In the search for new therapeutic targets for the treatment of alcoholism, more attention should be directed to drugs that act on relevant epigenetic modifications.

Better understanding of the effect of alcohol abuse in epigenetic alterations may help to identify targets for intervention which can potentially reverse alcohol-related pathological changes and behaviors. Fortunately, there are a number of drugs that target epigenetic enzymes; although originally developed for the treatment of specific cancers, application of these agents to psychiatric disorders, including alcohol and substance use disorders, may be possible (Grayson et al. 2010). Some of the molecules being tested in preclinical models are FDA-approved epigenetic-related medications such as suberoylanilide hydroxamic acid (SAHA), azacitidine, and decitabine; in some cases, these drugs are effective in diminishing alcohol-related behaviors in rodents. In the VTA of alcohol-preferring mice after binge drinking, there was decreased expression of genes that are involved in histone modification (Marballi et al. 2016). SAHA, trichostatin A (TSA), and other HDAC inhibitors also reduce drinking behavior (Jeanblanc et al. 2015; Legastelois et al. 2013; Simon-O’Brien et al. 2015; Warnault et al. 2013). GABA hyposensitivity of VTA dopaminergic neurons induced by chronic ethanol treatment is associated with increased HDAC2 and decreased acetylation on H3K9, and is reversible by HDAC inhibitors TSA and SAHA (Arora et al. 2013). DNMT inhibitor decitabine desensitized DA neurons in the VTA and blocked alcohol drinking behavior (Ponomarev et al. 2017). Increased drinking that is associated with increases in DNMT1 expression is reduced by DNMT inhibitors azacitidine and decitabine (Barbier et al. 2015; Warnault et al. 2013). Studies on these drugs suggest effective reversal of innate and manipulated deficits in epigenetic modification in rodent alcoholism models; the sites of action include but are clearly not limited to the VTA in these studies. Histone modifications in the VTA were also altered by other drugs of abuse such as toluene (Sanchez-Serrano et al. 2011) and morphine (Wang et al. 2014), while HDAC inhibitors have been shown to effectively reverse the morphine-induced changes in DA VTA activity (Authement et al. 2016).

It should be noted that some studies failed to reduce drinking by manipulating these same epigenetic factors. For example, both TSA and azacitidine facilitated voluntary drinking behavior after intermittent vapor exposure; administration of SAM, to increase methylation, reversed the effect of TSA and also reduced ethanol-induced ethanol consumption (Qiang et al. 2014); TSA also increased two-bottle choice drinking (Wolstenholme et al. 2011). There are numerous factors in which these two studies differ from those that showed reduction of drinking with HDAC and DNMT inhibitors, indicating that there are complexities that need to be recognized in the development and application of histone-modifying drugs for the treatment of alcoholism. Epigenetic modifications are dynamic processes that are likely to vary during different stages of the development of addiction and dependence, from acute intake to chronic use, with the additional complication of withdrawal from alcohol exposure at any of these stages. Additional factors in preclinical studies include the use of different species, different alcohol treatment protocols, and different durations of alcohol exposure. Differences also have been noted in epigenetic modification seen between human and animal models. For example, alcohol produces a decreased DNMT1 expression in alcohol-dependent human subjects (Ponomarev et al. 2012) while DNMT1 was increased in alcohol-dependent mice (Warnault et al. 2013) and rats (Barbier et al. 2015). Extensive preclinical studies are needed to define the proper therapies and conditions for treatment of human alcoholics.

Despite the limitations and challenges, the above discussed literature opens an intriguing possibility of using epigenetic modulators in alcoholism prevention and treatment. Some FDA-approved drugs were able to normalize epigenetic alterations in the VTA and regulate drinking behaviors in animal models. Restoring the balance in reward/aversion processes may be one aspect of these agents that appear to normalize at least some of the physiological processes altered by chronic alcohol exposure. This “repurposing” of FDA-approved anti-cancer drugs (which includes the ALK antagonist alectinib as well as most of the HDAC and DNMT inhibitors) for the treatment of alcoholism has the advantage of reducing the regulatory path to clinical usage (Tulisiak et al. 2017). With emerging new techniques, novel compounds might be possible in the near future to target more specific elements (such as more selective inhibitors of specific HDAC isoforms) of epigenetic regulatory pathways to overcome the complexity of epigenetic modification mechanism.

## Consideration of sex differences

Layered upon the complexity of the development of any effective treatments for alcohol use disorders (using epigenetic or more conventional strategies) is the issue of sex differences. Numerous studies have established significant sex differences in alcohol drinking. More men than women drink; however, this “gender gap” is declining as the prevalence of alcohol consumption is increasing in women (Keyes et al. 2008). Alcohol-induced impairments, such as impaired cognition, vary between males and females (Dudek and Phillips 1990; Jones and Whitfield 1995; Middaugh et al. 1992). Finally, women are more vulnerable to health risks associated with alcohol consumption such as liver disease and brain damage (Eagon 2010; Mann et al. 1992). Ethanol consumption in animal studies varies with species, with female rodents and male nonhuman primates generally drinking more (Lancaster and Spiegel 1992; Vivian et al. 2001). These sex differences may not be due to differing magnitudes of the same mechanisms, but could be discrete mechanisms. When seeking to restore the aversion/reward balance in the VTA, distinct strategies may be necessary for males and females. The observed sex differences in alcoholism stress the importance of understanding the mechanisms for addictive behaviors in both males and females.

A substantial number of studies have examined sex differences in dopaminergic neurotransmission. Evidence from both clinical and preclinical studies have identified inherent sex differences in dopaminergic systems. Clinical studies have demonstrated that striatal dopamine synthesis and DAT expression levels are higher in females compared to males (Laakso et al. 2002; Lavalaye et al. 2000; Mozley et al. 2001). Dopamine D_2_ receptor affinities are greater in men than in women (Pohjalainen et al. 1998). Sex-associated structural differences in the VTA may also partially contribute to differences in reward-seeking behaviors. There are a greater number of dopaminergic neurons and a larger VTA volume in females (McArthur et al. 2007a). The general shape of the VTA and localization of cells within the VTA is different between sexes (McArthur et al. 2007a); in males, dopaminergic neuronal subtypes were evenly distributed throughout the layers of the VTA, whereas in females, dopaminergic neuronal subtypes were more segregated in specific regions (McArthur et al. 2007a).

In addition to cytoarchitectural differences in dopamine brain areas, there are also differences in dopamine circuit composition between sexes. There are differences in the distribution of DA VTA neurons making up mesocortical pathways in males and females. In female rats, there is a larger proportion of VTA DA cells projecting to the prefrontal cortex than in males (Kritzer and Creutz 2008). Interestingly, in male rats, there are differences in the composition of androgen and estrogen receptors on DA VTA neurons that project to different brain regions, suggesting sex hormones may play distinct roles in modulating dopamine functions (Creutz and Kritzer 2004).

### Sex hormones and dopamine

Hormones play an important role in shaping dopaminergic neurotransmission and tone during prenatal and prepubertal development. Sex chromosomes and gonadal steroids play a major role in the development of the brain, including dopaminergic pathways and establishing sex differences (McCarthy and Arnold 2011). The fetal hormonal development of the brain in males is mediated, in part, by estrogens produced by the aromatization of testosterone (Arnold 2009; McCarthy et al. 2008; Wilson and Davies 2007). Testosterone is required during perinatal development to achieve normal basal dopamine content in the frontal cortex in males (Stewart and Rajabi 1994). There is also a role of estrogens for early brain development in females, suggesting differential expression of sex steroid receptors may coordinate sex differences in brain development (Bakker et al. 2003). During puberty, a critical time for the development of sex differences, sex hormones also drive sexual differentiation of the brain (Juraska et al. 2013; Sisk and Zehr 2005) and may actively modulate the development of dopaminergic systems (Kuhn et al. 2010). The development of sex differences in DA-driven behaviors are sensitive to environmental conditions during these two developmental periods (Gillies et al. 2014; Sisk and Zehr 2005), and exposure to alcohol during these crucial periods may alter the construction and refinement of dopamine reward pathways. Early drug exposure increases the likelihood for the development of abuse later in life (Grant and Dawson 1997). Premature pubertal development in girls is correlated with an earlier age of onset for alcohol consumption (Dick et al. 2000; Wilson et al. 1994). Although no causative relationship between premature pubertal development and alcohol consumption has been established, it would be interesting to investigate whether hormonal treatments for premature development in girls would also delay or prevent the onset of alcohol use. Even during early adulthood, the mesocortical system is still developing (Spear 2000), increasing the relevance of studying the interaction of early alcohol exposure and long-term alteration of DA VTA physiology and reward pathways.

In addition to their role in development, sex hormones may play important roles in modulating dopamine neurotransmission in adults. Both androgens and estrogens affect cortical function in men and women (Cherrier et al. 2003; Miller et al. 2006). Estradiol, the main form of estrogen in adult pre-menopausal females, is synthesized in the brains of both males and females (Do Rego et al. 2009; Pelletier 2010; Simpson and Jones 2006). Estradiol action in the brain is mediated by neuronal estrogen receptors ERα, ERβ, and the G protein-coupled estrogen receptor (GPER) (Levin and Hammes 2016). Both ERα and ERβ are expressed in the VTA (Creutz and Kritzer 2002; Kritzer 1997; Milner et al. 2010; Vanderhorst et al. 2005). In female rodents, estradiol modulates dopamine dynamics such as synthesis, receptor expression, and dopamine transporter quantities (Lammers et al. 1999; McArthur et al. 2007a; Pasqualini et al. 1995). Conversely, castration and hormonal treatments in male rodents had no effect on dopamine dynamics (Becker 1999; McArthur et al. 2007b). Dopamine dynamics are differently altered by sex hormones in males and females, suggesting differences in receptor activities or downstream factors. Estradiol levels fluctuate during the 4–6-day estrous cycle in rodents (Nilsson et al. 2015). Dopamine mesocortical transmission varies with the estrous cycle in naïve female rodents under basal conditions, and ethanol-induced activation of the mesocortical system also varies with the estrous cycle (Dazzi et al. 2007).

### Sex hormones and drug reward

Because of this hormonal influence on dopaminergic transmission, sex hormones play an important role in modulating drug reward. Psychostimulant drug reward is stronger in females (Becker and Hu 2008; Carroll et al. 2004; Lynch 2006). The rewarding effects of psychostimulant drugs vary along the hormonal cycle of women, with a heightened response during high estradiol levels (Caldu and Dreher 2007). Ovariectomy reduces the response to amphetamines, and supplementation restores the magnitude of the response (Becker 1999; Ohtani et al. 2001; Xiao and Becker 1994). Amphetamine responses are enhanced in high estradiol states of the estrous cycle in female rats (Becker 1999; Fink et al. 1996). Estradiol also has a role in modulating ethanol consumption. Ovariectomization of female rats reduces ethanol intake, and intake is restored by supplementation with estradiol (Ford et al. 2002a). Correlation with ethanol consumption at different stages of the estrous cycle of rodents is not as evident. While some studies show no differences in ethanol consumption at different estrous stages in rodents (Ford et al. 2002b; Priddy et al. 2017), others have observed such differences (Forger and Morin 1982; Roberts et al. 1998). This discrepancy could be due, in part, to technical challenges in the ability to capture drinking behaviors modulated by rapidly changing hormones in rodents. Hormone-dependent drug responses are ideally similar between high estrogen states in freely cycling and ovariectomized/estrogen-supplemented rodents. For example, we have shown that the sensitivity of DA VTA neurons to inhibition by dopamine and excitation by ethanol is enhanced in high estradiol states in naive freely cycling and ovariectomized female mice (Vandegrift et al. 2017).

An understanding of sex differences in dopaminergic reward systems is needed in order to develop therapies effective for both males and females. Some current treatments for alcoholism exhibit gender differences in effectiveness. One example of these gender differences is the response to naltrexone, an opiate antagonist that has been approved by the FDA to treat alcohol abuse. Naltrexone acts, at least in part, on the reward system (O’Malley et al. 1992; Volpicelli et al. 1992). Naltrexone can be used to treat cocaine and alcohol abuse but it has been a less successful treatment in females (Suh et al. 2008). Women experience more severe side effects of naltrexone, such as nausea (O’Malley et al. 2000); the heightened adverse effects of naltrexone in women reduce compliance and result in women discontinuing use more often than males. This is a clear example of a difference in the reward/aversion balance in females versus males with respect to the influence of opiadergic systems. Sex hormones modulate opioid systems (Piva et al. 1995), and this fact may underlie the sex differences undermining the use of naltrexone to treat females with alcohol use disorders. Investigating the mechanisms of alcohol abuse and the reactions to novel pharmacological interventions should be taken into consideration in both male and female subjects. In order to restore the reward/aversion balance, in the VTA and elsewhere in the brain, it may be necessary to implement treatment regimens based on different pharmacology in men and women.

## Summary and conclusions

The VTA is the source of dopamine for reward/reinforcement pathways in the brain, but it also supplies dopamine to brain areas primarily associated with other behavioral functions (e.g., hippocampus, amygdala). The VTA is important for evaluation of the salience of environmental stimuli, including both aversive and rewarding stimuli. Neuroadaptive changes in the VTA induced by chronic alcohol use may change the balance of alcohol effects on the VTA, emphasizing the rewarding effects of alcohol and diminishing the aversive components of alcohol action. The goal in developing therapeutics for the treatment of alcoholism, from the limited perspective of the VTA, may be to restore the reward/aversion balance with respect to alcohol action. This review covered several aspects of alcohol action in the VTA, and provided thoughts regarding treatment development. The pharmacology of ethanol in the VTA, and the neurotransmitters and pathways involved in the acute and chronic effects of alcohol, may provide strategies to counteract the imbalance in the VTA. Epigenetics may reveal mechanisms at the genomic level that could be exploited to reverse some of the neuroadaptation induced by alcohol abuse and restore the reward/aversion balance. Sex differences in VTA physiology and in pharmacological responses indicate that sex-specific therapies may be needed to restore reward/aversion balance in both men and women. Consideration of all of these VTA-related factors also should be relevant in overall approaches for the development of agents useful for the treatment of alcohol use disorders.
